# Radiation Countermeasure Gamma-Tocotrienol Inhibits Accumulation of Lipid Peroxidation Products in the Serum of Nonhuman Primates Exposed to Partial- or Total-Body Radiation—A Hallmark of Inhibition of Irradiation-Induced Ferroptosis?

**DOI:** 10.3390/ijms27083387

**Published:** 2026-04-09

**Authors:** Kamil Brzóska, Alana D. Carpenter, Sarah A. Petrus, Vijay K. Singh

**Affiliations:** 1 Centre for Radiobiology and Biological Dosimetry, Institute of Nuclear Chemistry and Technology, Dorodna 16, 03-195 Warsaw, Poland; 2 Division of Radioprotectants, Department of Pharmacology and Molecular Therapeutics, F. Edward Hébert School of Medicine, Uniformed Services University of the Health Sciences, Bethesda, MD 20814, USA; 3 Armed Forces Radiobiology Research Institute, Uniformed Services University of the Health Sciences, Bethesda, MD 20814, USA

**Keywords:** GT3, gamma-tocotrienol, ferroptosis, lipid peroxidation, radioprotection, partial-body irradiation, total-body irradiation

## Abstract

Gamma-tocotrienol (GT3) is one of the constituents of vitamin E that demonstrated significant radioprotective efficacy in murine and nonhuman primate (NHP) models. Considering the antioxidant activity of GT3 and its role in terminating lipid peroxidation, we hypothesize that mechanism of radioprotective effect of GT3 may involve the inhibition of irradiation-induced ferroptosis—a form of regulated cell death characterized by excessive, iron-dependent, peroxidation of lipids in cellular membranes. To test this hypothesis, the metabolomic and proteomic data from serum samples of GT3- or vehicle-treated NHPs exposed to 12 Gy (partial- or total-body) radiation was analyzed with focus on lipid peroxidation markers and proteins involved in iron metabolism. Four secondary lipid peroxidation products were identified including 4-oxo-2-nonenal (4-ONE), 4-hydroperoxy-2-nonenal (4-HPNE), 3,4-epoxynonanal (3,4-ENA), and trans-4,5-epoxy-(2E)-decenal (4,5-EDE). In vehicle-treated animals, their concentrations increased significantly as soon as 4 h after irradiation and then gradually declined. GT3 treatment mitigated this radiation-induced increase. In addition to lipid peroxidation products, similar patterns of change were observed for several polyunsaturated, monounsaturated, and saturated fatty acids as well as amino acids such as lysine and its derivatives. Taken together, these metabolomic changes suggest that irradiation induces cellular membrane damage through enhanced lipid peroxidation, while GT3 exerts a protective effect against this process. In addition, GT3 increased serum levels of haptoglobin and hemopexin—two plasma scavenger proteins that play complementary protective roles in iron and heme homeostasis. Although the present study does not conclusively demonstrate that GT3 mediates radioprotection via inhibition of ferroptosis, the data suggest that GT3 limits membrane damage and reduces susceptibility to ferroptosis by enhancing iron and heme scavenging. Further investigation into the interaction between GT3 and key components of ferroptosis following exposure to ionizing radiation is therefore warranted.

## 1. Introduction

Ferroptosis is a form of regulated cell death characterized by excessive, iron-dependent peroxidation of lipids in cellular membranes. This process results from oxidative stress, which arises due to disruptions in the antioxidant defense system, imbalances in cellular metabolism, or external factors such as ionizing radiation [1,2]. In fact, ferroptosis has been shown to contribute to the adverse effects of radiation, such as lung fibrosis [3] and death of granulocyte-macrophage hematopoietic progenitor cells caused by radiation-induced iron accumulation in the bone marrow [4]. Moreover, ferroptosis-promoting iron deposition in the bone marrow and spleen after total-body irradiation was observed in murine and nonhuman primate (NHP) models [5,6].

It is well-known that ferroptosis can be suppressed by antioxidants such as vitamin E that scavenges lipid peroxyl radicals to disrupt the propagation phase of the lipid peroxidation process [2]. One of the constituents of vitamin E is γ-tocotrienol (GT3) that demonstrated significant in vivo radioprotective activity in murine and NHP models [7,8]. It is currently under advanced development as a promising medical countermeasure for prophylaxis against acute radiation syndrome (ARS) following high dose partial- or total-body irradiation [9]. Several mechanisms responsible for GT3 radioprotective activity have been described including induction of high levels of granulocyte colony-stimulating factor (G-CSF) expression, which promotes survival and recovery of hematopoietic stem and progenitor cells [10], inhibition of 3-hydroxy-3-methylglutaryl-coenzyme A (HMG-CoA) reductase resulting in reduction in vascular peroxynitrite generation [11], upregulation of thrombomodulin—an anticoagulant with radioprotective efficacy [12], modulation of radiation-induced oxidative and nitrosative stress by CCAAT enhancer binding protein delta (CEBPD) upregulation [13], stabilization of RAD50 expression and decreased frequency of radiation-induced double-strand breaks and chromosomal aberrations [14].

Considering the antioxidant activity of vitamin E family members, including GT3, and their critical role in terminating lipid peroxidation, we hypothesize that an additional, as-yet-undescribed mechanism of GT3 radioprotective effect in vivo may involve the inhibition of radiation-induced ferroptosis. In the present study, we analyzed the metabolomic and proteomic data from serum of NHPs irradiated with high lethal doses of radiation in both partial- and total-body models. We focused our analysis on highly reactive aldehydes which are products of non-enzymatic peroxidation of polyunsaturated fatty acids (PUFAs) and may therefore serve as markers of lipid peroxidation triggered by radiation-induced oxidative stress. We also analyzed other metabolites whose concentration changes following irradiation and GT3 treatment showed a pattern similar to that of the aldehydes. Furthermore, we analyzed the expression of proteins important for iron metabolism and, consequently, ferroptosis.

## 2. Results

### 2.1. Lipid Peroxidation Products

**Figure 1:**
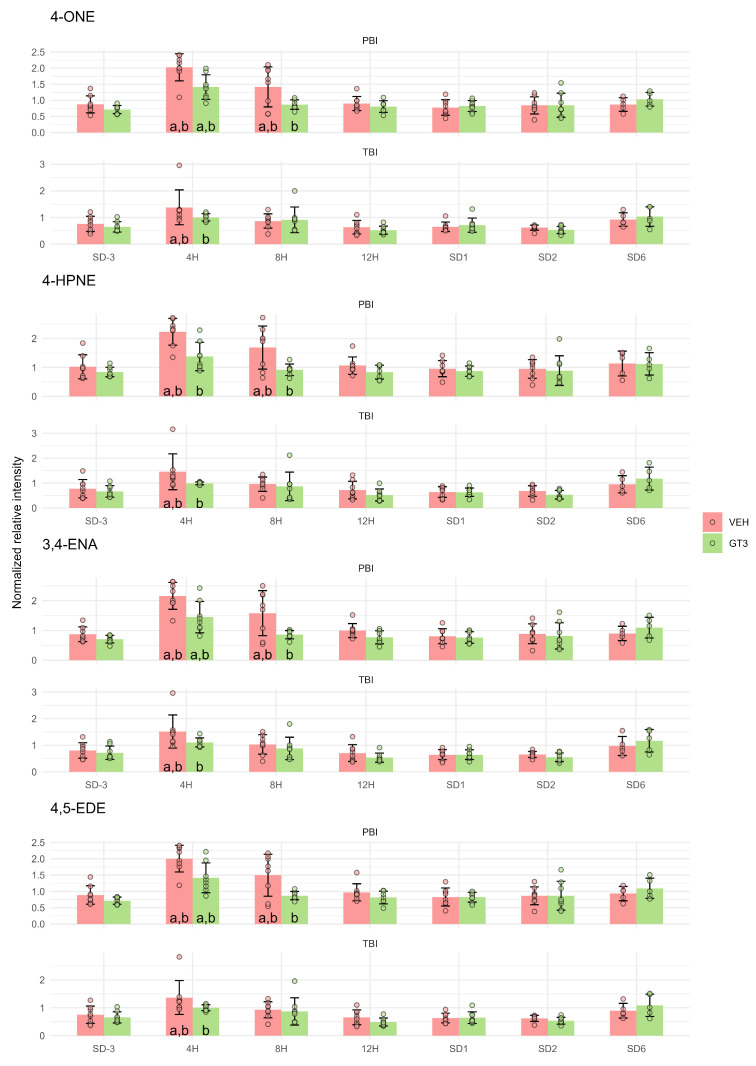
Serum levels of 4-oxo-2-nonenal (4-ONE), 4-hydroperoxy-2-nonenal (4-HPNE), 3,4-epoxynonanal (3,4-ENA), and trans-4,5-epoxy-(2E)-decenal (4,5-EDE) in NHPs subjected to partial- or total-body irradiation. Bars represent mean values, whiskers indicate standard deviations, and circles correspond to individual data points. “a” denotes a statistically significant difference compared with the pre-irradiation sample; “b” denotes a statistically significant difference between vehicle-treated and GT3-treated animals.

The metabolomic dataset generated during the analysis of blood serum from GT3- and vehicle-treated NHPs subjected to partial- or total-body irradiation [15] was examined for lipid peroxidation products that could indicate ongoing radiation-induced ferroptosis. Four secondary lipid peroxidation products that derive from the breakdown of polyunsaturated fatty acid hydroperoxides (primary products of peroxidation) were identified. These include 4-oxo-2-nonenal (4-ONE), 4-hydroperoxy-2-nonenal (4-HPNE), 3,4-epoxynonanal (3,4-ENA), and trans-4,5-epoxy-(2E)-decenal (4,5-EDE). All of them showed similar temporal metabolic patterns following irradiation and similar modulation by GT3 treatment (Figure 1).

In vehicle-treated animals, the concentrations of these metabolites increased approximately twofold 4 h after PBI. Subsequently, levels gradually declined. At 8 h post-PBI, they remained significantly higher than pre-irradiation levels but were lower than at 4 h. At later time points, metabolite concentrations returned to the pre-irradiation level. GT3 treatment mitigated the radiation-induced increase in reactive aldehydes, as the elevation observed at 4 h post-PBI was significantly reduced compared with vehicle-treated animals. Furthermore, GT3-treated animals showed a faster return to baseline levels, with metabolite concentrations at 8 h post-PBI not differing significantly from pre-irradiation values.

A similar, though less pronounced, pattern was observed following TBI. In vehicle-treated animals, the increase in reactive aldehydes was modest and evident only at 4 h post-irradiation. Nonetheless, the protective effect of GT3 was apparent, as no significant changes in metabolite concentrations were observed in GT3-treated animals.

### 2.2. Polyunsaturated Fatty Acids and Their Derivatives

To place the findings on lipid peroxidation products within the broader context of metabolomic changes caused by ionizing radiation, we analyzed the same dataset to identify other metabolites showing similar patterns of change after irradiation and GT3 treatment.

**Figure 2:**
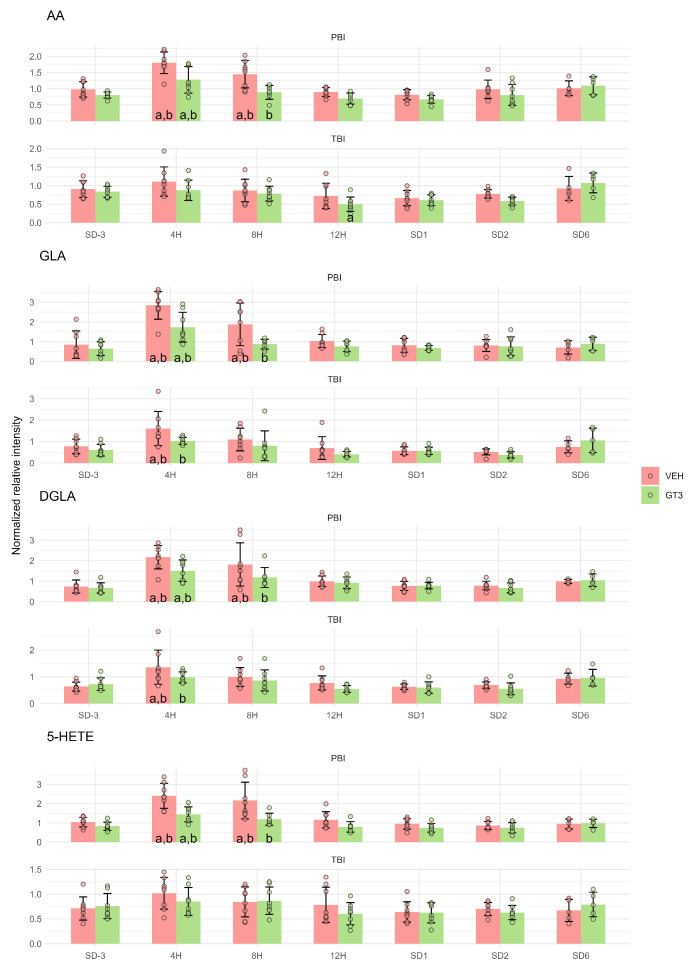
Serum levels of arachidonic acid (AA), (6Z,9Z,12Z)-octadecatrienoic acid (γ-linolenic acid—GLA), (8Z,11Z,14Z)-icosatrienoic acid (dihomo-γ-linolenic acid—DGLA), and 5-hydroxyeicosatetraenoic acid (5-HETE) in NHPs subjected to partial- or total-body irradiation. Bars represent mean values, whiskers indicate standard deviations, and circles correspond to individual data points. “a” denotes a statistically significant difference compared with the pre-irradiation sample; “b” denotes a statistically significant difference between vehicle-treated and GT3-treated animals.

Interestingly, several PUFAs that are susceptible to peroxidation also increased in serum in a manner similar to their peroxidation products. These included arachidonic acid (AA), (6Z,9Z,12Z)-octadecatrienoic acid (γ-linolenic acid—GLA), (8Z,11Z,14Z)-icosatrienoic acid (dihomo-γ-linolenic acid—DGLA), and 5-hydroxyeicosatetraenoic acid (5-HETE). GLA and DGLA are intermediates in the enzymatic conversion of linoleic acid (LA) to AA, while 5-HETE is formed through the enzymatic oxidation of AA by ALOX5 and functions as a pro-inflammatory mediator. All these metabolites displayed a similar metabolomic profile to lipid peroxidation products, with peak concentrations occurring 4 h after PBI and significant attenuation following GT3 treatment (Figure 2). These effects were weaker after TBI, and for AA and 5-HETE, no significant changes were observed in response to either irradiation or GT3 treatment (Figure 2).

**Figure 3:**
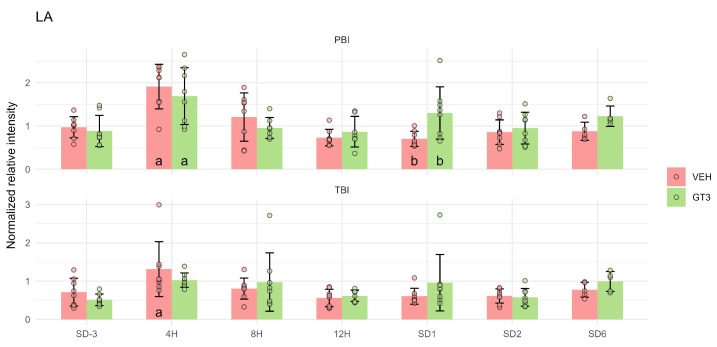
Serum level of linoleic acid (LA) in NHPs subjected to partial- or total-body irradiation. Bars represent mean values, whiskers indicate standard deviations, and circles correspond to individual data points. “a” denotes a statistically significant difference compared with the pre-irradiation sample; “b” denotes a statistically significant difference between vehicle-treated and GT3-treated animals.

Interestingly, LA exhibited a distinct metabolic pattern compared to other PUFAs and their peroxidation products. Its concentration increased significantly 4 h after PBI, but GT3 treatment had no notable effect at that time point. The only significant influence of GT3 on LA levels was observed 1 d after PBI, when GT3-treated animals showed higher LA concentrations than vehicle-treated controls. As with other metabolites, these effects were considerably weaker following TBI compared to PBI (Figure 3).

### 2.3. Monounsaturated and Saturated Fatty Acids

**Figure 4:**
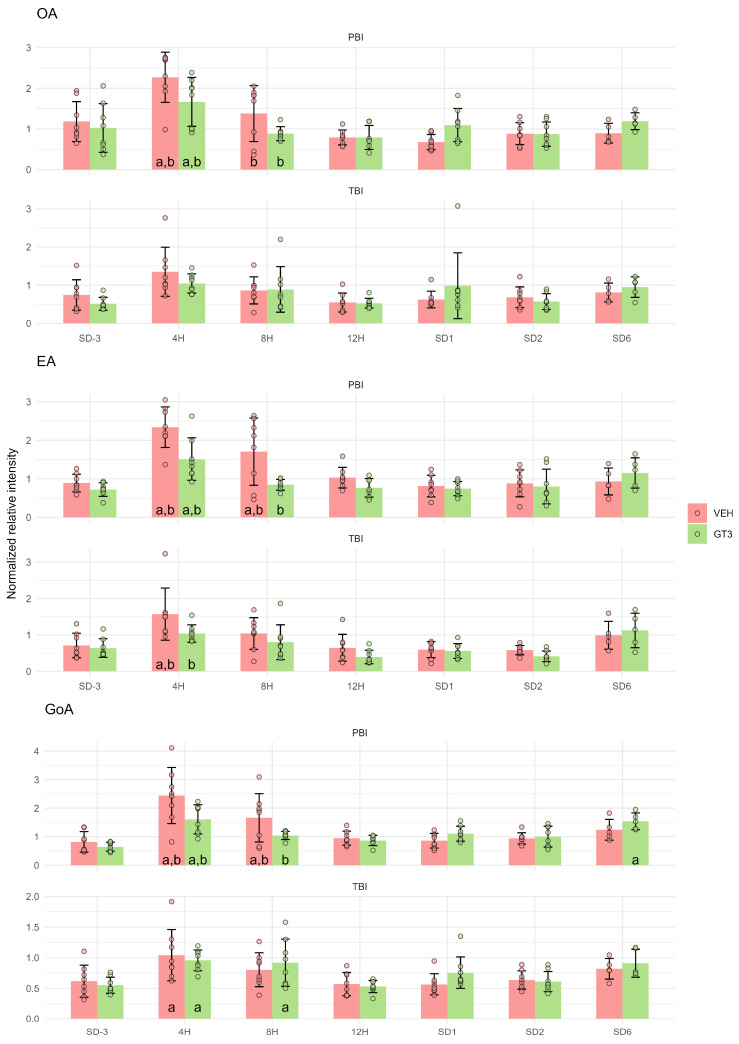
Serum levels of (9Z)-Octadecenoic acid (OA), (9E)-Octadecenoic acid (EA), and gondoic acid (GoA) in NHPs subjected to partial- or total-body irradiation. Bars represent mean values, whiskers indicate standard deviations, and circles correspond to individual data points. “a” denotes a statistically significant difference compared with the pre-irradiation sample; “b” denotes a statistically significant difference between vehicle-treated and GT3-treated animals.

**Figure 5:**
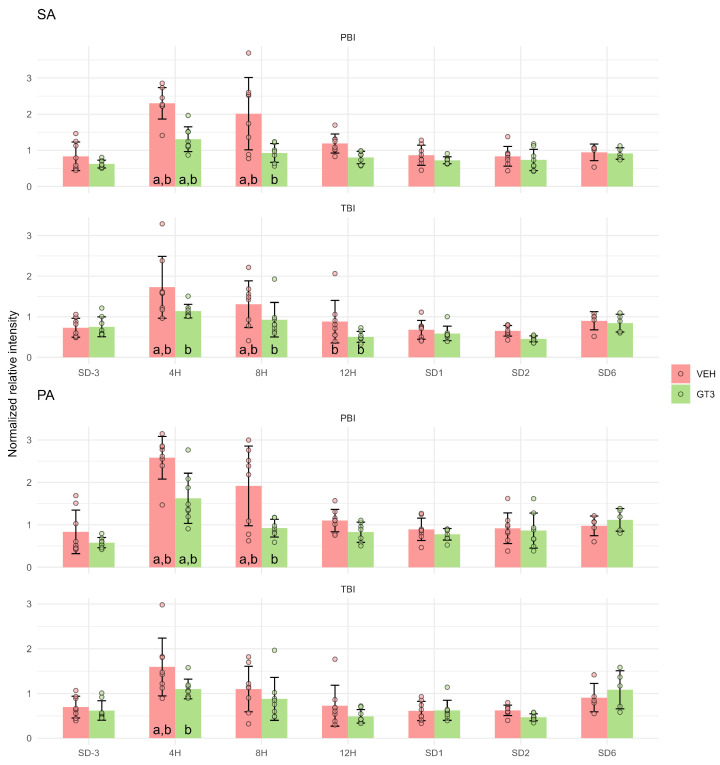
Serum levels of octadecanoic acid (stearic acid—SA) and hexadecanoic acid (palmitic acid—PA) in NHPs subjected to partial- or total-body irradiation. Bars represent mean values, whiskers indicate standard deviations, and circles correspond to individual data points. “a” denotes a statistically significant difference compared with the pre-irradiation sample; “b” denotes a statistically significant difference between vehicle-treated and GT3-treated animals.

Monounsaturated fatty acids, including gondoic acid (GoA), (9Z)-Octadecenoic acid (OA), (9E)-Octadecenoic acid (EA) (Figure 4), exhibited the same metabolic pattern as peroxidation products and polyunsaturated fatty acids. Similarly, two major saturated fatty acids found in cell membranes, hexadecanoic acid (palmitic acid—PA) and octadecanoic acid (stearic acid—SA), showed comparable responses (Figure 5). All these fatty acids peaked 4 h after irradiation, with levels reduced by GT3 treatment. As observed previously, these effects were less pronounced following TBI than PBI.

Overall, the simultaneous increase in polyunsaturated, monounsaturated, and saturated fatty acids shortly after irradiation appears to reflect irradiation-induced damage to cellular membranes. This damage is closely linked to ongoing lipid peroxidation, as indicated by elevated levels of 4-ONE, 4-HPNE, 3,4-ENA, and 4,5-EDE.

### 2.4. Amino Acids

**Figure 6:**
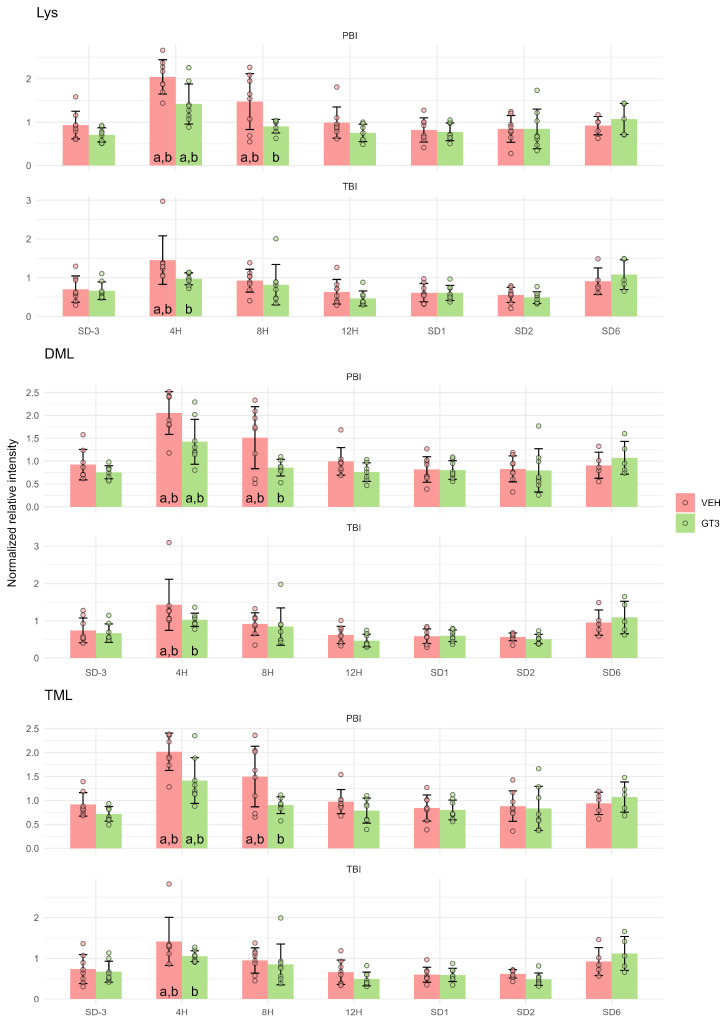
Serum levels of L-Lysine (Lys) and its methylated forms: N6,N6,N6-Trimethyl-L-lysine (TML) and Protein N6,N6-dimethyl-L-lysine (DML) in NHPs subjected to partial- or total-body irradiation. Bars represent mean values, whiskers indicate standard deviations, and circles correspond to individual data points. “a” denotes a statistically significant difference compared with the pre-irradiation sample; “b” denotes a statistically significant difference between vehicle-treated and GT3-treated animals.

Ongoing cell and tissue damage was further evidenced by increased levels of the amino acid L-lysine (Lys) and its methylated derivatives, N6,N6,N6-trimethyl-L-lysine (TML) and protein N6,N6-dimethyl-L-lysine (DML). These changes suggest enhanced protein turnover and tissue injury. All three metabolites followed the same pattern as observed for the fatty acids described above, with peak concentrations at 4 h after irradiation and marked attenuation following GT3 treatment (Figure 6).

### 2.5. Haptoglobin and Hemopexin

The detection of lipid peroxidation products confirms that oxidative damage to cellular membrane lipids occurred after irradiation. While such damage can be associated with ferroptosis, it may also arise independent of this process. Because ferroptosis is defined by its dependence on iron, we further examined serum proteomic data from the same experimental cohort to assess the impact of GT3 on iron metabolism. This analysis focused on proteins involved in iron regulation and revealed significant effects of GT3 treatment on two key proteins: haptoglobin (HP) and hemopexin (HPX).

**Figure 7:**
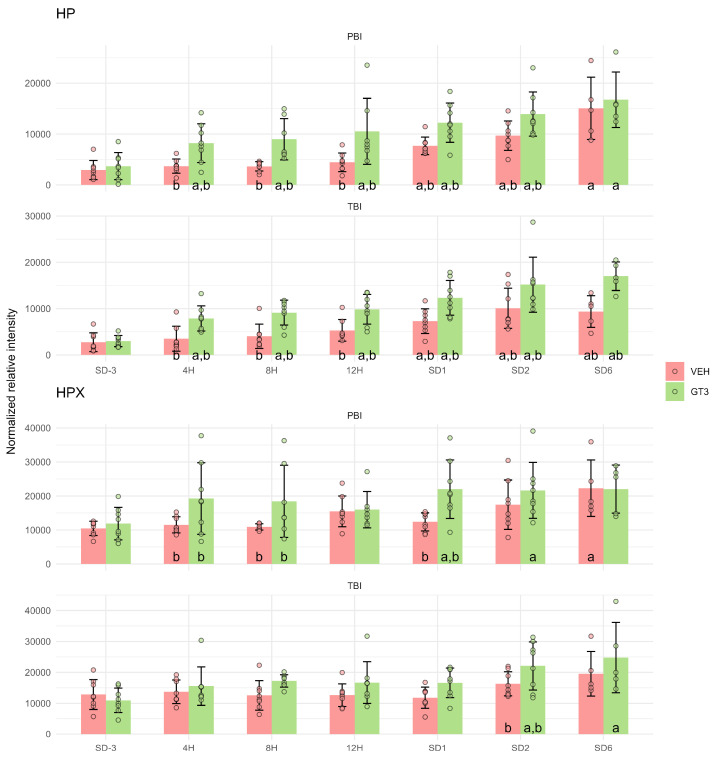
Serum levels of haptoglobin (HP) and hemopexin (HPX) in NHPs subjected to partial- or total-body irradiation. Bars represent mean values, whiskers indicate standard deviations, and circles correspond to individual data points. “a” denotes a statistically significant difference compared with the pre-irradiation sample; “b” denotes a statistically significant difference between vehicle-treated and GT3-treated animals.

Haptoglobin levels were markedly elevated in GT3-treated animals compared with vehicle-treated controls, with increases evident as early as 4 h after irradiation. This difference persisted up to 2 d post-irradiation in the PBI model and up to 6 d in the TBI model. In both models, HP levels in GT3-treated animals were significantly higher than in pre-irradiation samples from 4 h post-irradiation onward, whereas vehicle-treated animals showed no significant increase until 1 d post-irradiation (Figure 7).

Hemopexin displayed a similar GT3-induced upregulation, though the effect was less pronounced, particularly following TBI (Figure 7).

In summary, the observed increase in lysine and its derivatives, along with polyunsaturated, monounsaturated, and saturated fatty acids shortly after irradiation, reflects cellular and tissue damage that is associated with lipid peroxidation, as evidenced by elevated levels of its byproducts. Treatment with GT3 attenuates this damage and at the same time increases the serum levels of proteins involved in iron homeostasis.

## 3. Discussion

The primary products of lipid peroxidation are lipid hydroperoxides, which subsequently decompose into a range of aldehydes as secondary products. Among these, malondialdehyde (MDA) and 4-hydroxynonenal (4-HNE) are the most widely studied and commonly used biomarkers of lipid peroxidation [16]. Using the untargeted metabolomic approach applied to generate datasets analyzed in the present study, neither MDA nor 4-HNE was detected in the serum of NHPs subjected to TBI or PBI. However, several other aldehydes originating from lipid peroxidation were identified, including 4-ONE, 4-HPNE, 3,4-ENA, and 4,5-EDE. Importantly, 4-HPNE is a direct precursor of 4-HNE and 4-ONE, and such aldehydes are considered major contributors to the cytopathological effects associated with oxidative stress [17,18,19].

To the best of our knowledge, none of these four aldehydes has previously been investigated as a potential biomarker of radiation-induced damage relevant to biological dosimetry or development of radioprotective agents. The usefulness of these compounds as biomarkers may, however, be constrained by their temporal profile: their concentrations peak as early as 4 h after exposure and return to baseline levels by 12 h post-exposure. This kinetic pattern may be advantageous in applications focused on early radiation effects, where rapid assessment is required. Conversely, such early responses are often underrepresented, as these early time points are not consistently included in experimental designs, particularly in animal studies.

It should be noted that PBI consistently resulted in more pronounced effects than TBI. Although this finding is counterintuitive and cannot be fully explained by the available data, it is likely attributable to differences in radiation quality and exposure conditions, specifically the use of LINAC-generated X-rays for PBI and cobalt-60 γ-rays for TBI, and/or differences in dose rates (1.3 Gy/min for PBI versus 0.6 Gy/min for TBI), as discussed in previous publications [15,20].

The present analysis focused on aldehydes generated through lipid peroxidation to better elucidate the mechanisms underlying the radioprotective activity of GT3. The decreased levels of lipid peroxidation products observed in the serum of irradiated animals treated with GT3 provide clear evidence of a direct antioxidant effect. This effect complements previously described mechanisms of GT3 action, such as the induction of G-CSF expression [9]. Although lipid peroxidation can be associated with ferroptosis, it is not, by itself, indicative of this process. Ferroptosis is a regulated form of cell death that is dependent on iron and characterized by the accumulation of lipid peroxides due to impaired antioxidant defenses [21].

Studies in both mouse and NHP models have demonstrated that radiation-induced hemolysis of red blood cells leads to the release of iron and its subsequent accumulation in tissues including the bone marrow, spleen, liver, heart, and intestines [4,5,6,22,23,24,25]. This increased iron burden has been linked to tissue dysfunction and is thought to contribute to ferroptosis [4,6,22,24]. NHP tissues collected at the end of the experiment reported in the present study were examined histopathologically, revealing tissue damage across multiple major organ systems [26]. However, this analysis did not assess iron accumulation or ferroptosis markers.

To assess the relevance of radiation-induced iron release to the observed increases in lipid peroxidation products, the temporal relationship between these events must be considered. In NHPs exposed to 5.8–8.5 Gy TBI, iron accumulation in the bone marrow and spleen was detected 4–15 d after exposure [5]. Similarly, in a mouse model receiving 7.9 Gy TBI, iron accumulation in the bone marrow followed the kinetics of red blood cell hemolysis, with approximately tenfold increase in bone marrow iron observed 14–21 d post-irradiation, coinciding with the nadir of red blood cell counts [23]. In mice exposed to a sublethal dose of 6.85 Gy, iron accumulation in the spleen occurred 7–14 d after irradiation and was associated with the induction of apoptosis and possibly ferroptosis [6]. In contrast, other studies have reported earlier changes in iron levels. Serum iron was significantly elevated within 1 d following exposure to 7 Gy total-body gamma irradiation in mice [27,28]. In addition, Zhang et al. observed iron accumulation in the murine bone marrow as early as 1 d after exposure to 4 or 8 Gy TBI, with peak levels occurring on d 3 post-irradiation [4]. This iron accumulation was primarily attributed to increased hemosiderin formation resulting from irradiation-induced bleeding in the bone marrow and led to ferroptosis of granulocyte-macrophage hematopoietic progenitor cells. The use of ferroptosis inhibitors has been shown to improve survival in irradiated mice. Notably, this protective effect was time dependent, with greater improvements observed at later administration of the drug. For example, administration of ferrostatin-1 at 72 h after irradiation resulted in superior survival outcomes compared with administration at 48 or 24 h post-exposure [4].

Overall, the studies discussed above indicate that exposure to high doses of radiation leads to iron deposition in tissues that are not typically involved in iron storage, such as bone marrow and spleen. This process may represent a secondary mechanism of radiation-induced tissue injury that involves the induction of ferroptotic cell death occurring days after irradiation. In contrast, in the present study, lipid peroxidation products were detected in the blood serum as early as 4 h after irradiation. Therefore, these early changes are unlikely to be directly related to ferroptosis driven by radiation-induced iron accumulation that develops days later. Instead, the observed lipid peroxidation products most likely result from the immediate damage to cellular membranes caused by irradiation. This early lipid peroxidation may, however, trigger an initial wave of ferroptosis that is independent of the later phase associated with iron accumulation. Whether this early lipid peroxidation progresses to ferroptotic cell death likely depends on the cellular context, including labile iron availability and the expression of proteins involved in iron metabolism and oxidative stress response. Further in vitro and in vivo studies are needed to determine whether this early wave of ferroptosis occurs and, if so, under which conditions, as well as to clarify the role of GT3 in this context.

Lei et al. proposed that radiation induces lipid peroxidation through at least two parallel mechanisms. First, radiation-generated reactive oxygen species directly promote lipid peroxidation. Second, radiation increases ACSL4 expression, which is essential for the synthesis of polyunsaturated fatty acids—containing phospholipids (PUFA-PLs)—that are particularly susceptible to peroxidation [29]. In the present study, the first mechanism appears to be the dominant contributor to early lipid peroxidation. The second mechanism requires time for ACSL4 expression to increase and for susceptible lipid species to accumulate, making it less relevant to the immediate cellular response to irradiation. However, this pathway may become more important in settings such as fractionated radiotherapy, where elevated levels of PUFA-PLs could enhance susceptibility to lipid peroxidation following subsequent radiation exposures. It may also play a greater role in long-term radiation effects rather than in the early events that determine initial cell survival or death.

It should be noted that the transient increase in lipid peroxidation products, which peaked 4 h after irradiation, was accompanied by changes in other metabolites exhibiting a similar temporal pattern. These included polyunsaturated (Figure 2), monounsaturated (Figure 4), and saturated (Figure 5) fatty acids, as well as amino acids such as Lys, TML, and DML (Figure 6). Taken together, these findings are consistent with extensive cellular damage following irradiation, involving lipid peroxidation but not limited to this process alone. Importantly, this damage was markedly attenuated by GT3 treatment, as evidenced by the suppression of irradiation-induced increases in these metabolites.

An important question is whether these effects can be attributed to specific cell types or tissues. Because tocotrienols, including GT3, have been reported to preferentially accumulate in endothelial cells [30], it is reasonable to hypothesize that the metabolic changes observed at 4 h after irradiation reflect radiation-induced endothelial cell damage that is mitigated by GT3. Further studies are required to test this hypothesis.

The effect of GT3 on iron metabolism is supported by its ability to increase serum levels of haptoglobin and, to a lesser extent, hemopexin—two plasma scavenger proteins that play complementary protective roles in iron and heme homeostasis, particularly during intravascular hemolysis [31]. The primary function of these proteins is to prevent iron loss and to limit iron-mediated oxidative damage. Haptoglobin binds free hemoglobin released during red blood cell hemolysis, whereas hemopexin scavenges free heme released upon hemoglobin dissociation or from other heme-containing proteins. Notably, both proteins were upregulated as early as 4 h after irradiation in GT3-treated animals (Figure 7). This early response suggests that GT3 may reduce lipid peroxidation and ferroptosis not only through direct antioxidant activity, but also indirectly by enhancing the clearance of free hemoglobin and heme. Distinguishing between lipid peroxidation as a general oxidative process and lipid peroxidation occurring within the context of ferroptosis is essential to correctly attribute the radioprotective effect—whether the agent acts as a general antioxidant limiting membrane damage, or specifically interferes with ferroptotic signaling and execution—since these mechanisms imply different targets, temporal dynamics, and implications for cell survival and tissue protection [32]. Collectively, the data indicates that GT3 likely exerts radioprotective effects through both pathways: by limiting membrane damage via its antioxidant properties and by reducing susceptibility to ferroptosis through enhanced iron and heme scavenging.

One of the major challenges in studying ferroptosis is the lack of well-established biomarkers that allow its precise and definitive assessment, particularly in vivo [33,34]. 4-ONE, 4-HPNE, 3,4-ENA, and 4,5-EDE are lipid peroxidation products derived from the peroxidation of polyunsaturated fatty acids. Although they may reflect lipid peroxidation processes associated with ferroptosis, they are not specific markers of this form of cell death. Ferroptosis is uniquely defined by its dependence on iron-catalyzed peroxidation of PUFA-PLs. While levels of these markers may increase under such conditions, their presence alone does not provide definitive evidence of ferroptosis. To reliably identify ferroptosis, these lipid peroxidation products should be evaluated in combination with more specific indicators, such as loss of GPX4 activity, iron dependence, and sensitivity to ferroptosis inhibitors. Accordingly, the present study cannot conclusively demonstrate that the radioprotective effects of GT3 are mediated through inhibition of ferroptosis. Rather, the findings suggest this possibility and support the need for further investigation.

Future research should include both additional in vivo studies and targeted in vitro experiments to better define the relationship between GT3 and key components of ferroptosis, including iron metabolism, GPX4 and ACSL4 activity, glutathione availability, and lipid peroxidation at the cellular level. Such studies should also address the mechanisms underlying GT3-mediated upregulation of haptoglobin and hemopexin. This kind of investigation may also contribute to the identification of more specific biomarkers of ferroptosis, particularly in the context of radiation-induced injury in vivo.

## 4. Materials and Methods

Detailed information concerning the experimental setup, materials and methods employed for sample preparation and analysis can be found in earlier publications [15,20]. For conciseness, the most pertinent information regarding the materials and methods is presented briefly below.

### 4.1. Animals

A total of 32 rhesus macaques (*Macaca mulatta*, Chinese sub-strain, 14 males and 18 females, 3.5–5.5 years of age, weighing 3.84–5.6 kg) were used. The animals were obtained from the National Institutes of Health Animal Center (NIHAC, Poolesville, MD, USA) or from an approved NHP vendor (PrimeGen, Hines, IL, USA). All animals were maintained at BIOQUAL, Inc. (Rockville, MD, USA), a facility accredited by the Association for Assessment and Accreditation of Laboratory Animal Care (AAALAC)-International. Animal procedures were approved (Protocol # 18-060 approved on 27 June 2018) by the Institutional Animal Care and Use Committee (IACUC, BIOQUAL, Inc.) and the Department of War Animal Care and Use Review Office. The Guide for the Care and Use of Laboratory Animals was strictly adhered to throughout the study [35]. Out of eight NHPs in each experimental group, three were euthanized on day 4 and five on day 7 post-irradiation with EUTHASOL (active ingredients pentobarbital sodium and phenytoin sodium, Virbac AH Inc., Fort Worth, TX, USA). Prior to pentobarbital sodium administration, animals were sedated using ketamine hydrochloride injection (Zoetis, Inc., Kalamazoo, MI, USA; 100 mg/mL, 5–15 mg/kg, intramuscularly).

### 4.2. Drug Preparation and Administration

GT3 (50 mg/mL) and the olive oil-based vehicle formulation were acquired from Callion Pharma, LLC (Jonesborough, TN, USA). A 37.5 mg/kg dose of GT3 or olive oil vehicle was prepared and administered to each animal 24 h prior to radiation exposure based on individual body weights. Pharmacokinetic parameters of GT3 in rhesus macaques were reported in an earlier publication and for the 37.5 mg/kg dose, demonstrated a maximum blood plasma concentration (C_max_) of 1142 ng/mL, the time at which C_max_ is obtained (T_max_) of 12h, half-life (T_1/2_) of 40.9 h, and mean retention time (MRT) of 11.4 d [8].

### 4.3. Radiation Exposure

Details of the irradiation procedure can be found in an earlier publication [15]. Briefly, for partial-body irradiation (PBI) NHPs were exposed to a 12 Gy dose using a 4 MV photon beam from an Elekta Infinity clinical linear accelerator (LINAC) with a dose rate of approximately 1.3 Gy/min. To spare 5% of the bone marrow, the field positioning was adjusted to extend from the top of the skull to the knee, excluding the tibia, ankles, and feet of the animals from radiation exposure. For total-body irradiation (TBI) the animals were exposed to a 12 Gy dose at a dose rate of 0.6 Gy/min using a high level ^60^Co gamma irradiator. Prior to irradiation, the NHPs were administered 10–15 mg/kg of ketamine hydrochloride (Zoetis, Inc., Kalamazoo, MI, USA; 100 mg/mL) through an intramuscular injection as anesthetic agent for sedation.

### 4.4. Blood Samples Collection

Blood was collected from the saphenous vein in serum-separation tubes, allowed to clot for at least 30 min, and centrifuged for 10 min at 400× *g*. Serum was isolated and stored at −70 °C until analysis. Blood samples were collected 3 d prior to irradiation as well as 4, 8, 12 h, 1 d, 2 d, and 6 d post-exposure.

### 4.5. Metabolomic and Proteomic Analyses

Metabolomic profiling was performed using UPLC-QTOF-MS. Metabolites were extracted from serum using a solvent mixture composed of methanol, isopropanol, water, and internal standards, followed by protein precipitation with acetonitrile. Centrifugation was performed to collect supernatants for analysis. Samples were then run on a UPLC system coupled to a Xevo G2 QTOF mass spectrometer using either a BEH or CSH C18 column for metabolomics or lipidomics, respectively. Mass spectrometry was performed in both positive and negative ionization modes, with regular injections of pooled quality control samples to monitor data consistency and instrument performance. Full details of metabolomic analysis can be viewed in earlier publication [15].

Proteomic profiling was performed by means of liquid chromatography-tandem mass spectrometry (LC-MS). Details on sample preparation for LC-MS and data acquisition can be found in the earlier publication [20].

### 4.6. Statistical Analysis

Linear mixed models (LMMs) were utilized to analyze the effects of GT3 treatment and time after irradiation on metabolite and protein concentration in serum. The mixed function from the afex R package version 1.5-0 [36] was used to fit the models, with NHP ID (subject) specified as a random intercept to account for the non-independence of repeated measures within each animal. The fixed effects included the main effects of treatment (GT3 or vehicle) and time after irradiation, as well as their interaction. Post hoc pairwise comparisons were performed using the emmeans R package version 1.11.2-8 [37], with Tukey method for time within treatment and the Bonferroni method for treatment within time. PBI and TBI data were analyzed separately. The significance level of *p* < 0.05 was used for all statistical tests.

## Data Availability

Proteomics and metabolomics data have been deposited to the Dryad Digital Repository. The proteomics data is available at (https://doi.org/10.5061/dryad.9ghx3ffx0) (last accessed on 6 April 2026) and the metabolomics data is available at (https://doi.org/10.5061/dryad.k3j9kd5pr) (last accessed on 6 April 2026).
